# Pancreaticoduodenal arterial hemorrhage following blunt abdominal trauma treated with transcatheter arterial embolization: Two case reports

**DOI:** 10.1097/MD.0000000000022531

**Published:** 2020-10-02

**Authors:** Yeongtae Park, Yook Kim, Jisun Lee, Bum S. Cho, Jin Y. Lee

**Affiliations:** a Department of Radiology, Chungbuk National University Hospital; b Department of Radiology, College of Medicine, Chungbuk National University; c Department of Trauma Surgery, Chungbuk National University Hospital, Cheongju, Republic of Korea.

**Keywords:** blunt trauma, case report, pancreaticoduodenal hemorrhage, transcatheter arterial embolization

## Abstract

**Rationale:**

Although surgery has been the standard treatment for pancreaticoduodenal trauma because of the complex anatomical relation of the affect organs, transcatheter arterial embolization (TAE) has recently been introduced as a safe and effective treatment. However, TAE for pancreaticoduodenal arterial hemorrhage (PDAH) can be challenging because it is difficult to localize the involved artery and to embolize the bleeding completely due to the abundant collateral channels of the pancreaticoduodenal artery (PDA).

**Patient concerns:**

Herein, we report 2 cases of PDAH that occurred after falling down in case 1 and a pedestrian traffic accident in case 2.

**Diagnoses:**

Multidetector computed tomography scan revealed massive retroperitoneal hematoma with active extravasation of contrast media from the PDA without any duodenal perforation or advanced pancreatic injury in both patients.

**Interventions:**

All patients were successfully treated using only TAE with a combination of microcoils and *n*-butyl cyanoacrylate (NBCA) in case 1, and only NBCA in case 2.

**Outcomes:**

There was no complication such as duodenal ischemia or pancreatitis. Laparotomy was not needed after TAE.

**Lessons:**

In selective PDAH cases, TAE may be a reasonable alternative to emergency laparotomy. It is expected that a careful and repetitive approach, based on complete angiography and embolization with a permanent liquid embolic agent such as NBCA could increase the success rate of TAE.

## 1 Introduction

Pancreaticoduodenal injury after blunt abdominal trauma is relatively rare (5% of all abdominal injuries).^[1]^ However, it is associated with intra-abdominal or retroperitoneal hemorrhages in the acute phase and has the potential for late complications such as intestinal ischemia or perforation. Recently, transcatheter arterial embolization (TAE) has been used as a safe and effective method to control intra-abdominal hemorrhage from hepatic, splenic, or renal injuries.^[2]^ In contrast, due to the complex anatomical position of the duodenum, pancreas, biliary tract, and major vessels, usually surgery is required to treat pancreaticoduodenal trauma.^[3]^ Therefore, management of pancreaticoduodenal arterial hemorrhage (PDAH) with TAE following blunt abdominal trauma is rare. However, surgical treatment is associated with significant morbidity because of surgery-related complications.^[4]^ Therefore, in the case of isolated PDAH without any major organ or intestinal injury, TAE should be considered as the first treatment approach. However, TAE for the pancreaticoduodenal artery (PDA) is technically challenging and sometimes impossible as it is difficult to predict the origin and to localize the artery on angiography and embolize the involved artery completely due to the abundant vascular supply and multiple potential collateral channels.^[5]^ Herein, we present 2 cases of isolated PDAH after blunt abdominal trauma that were successfully treated using TAE.

## 2 Case presentation

### 2.1 Case 1

**Figure 1:**
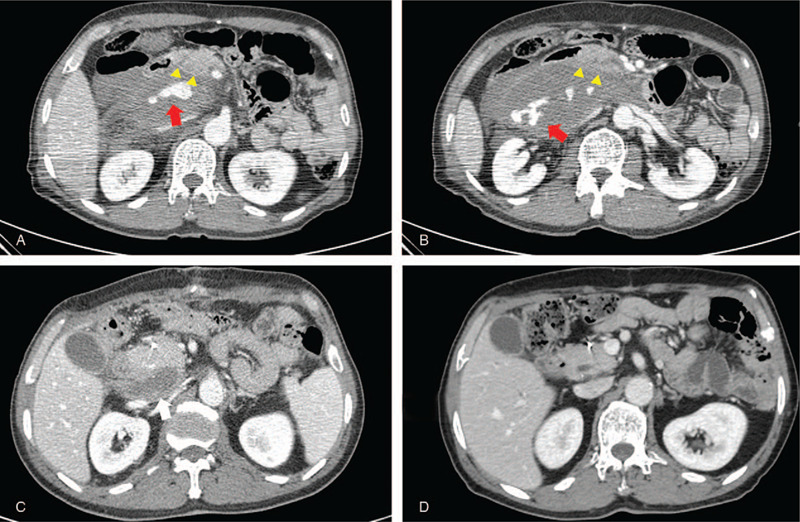
Case 1: a 64-year-old man was admitted to our trauma center after falling down from a height of 4 m. A, Multidetector computed tomography (MDCT) scan revealed as large amount of retroperitoneal hematoma at the posterior aspect of the pancreas head with internal extravasation of contrast media suggesting active bleeding (red arrow). Although there was a subtle low-density lesion suggesting mild contusion at the head of the pancreas, the shape of pancreas (yellow arrow head) and biliary duct were relatively well-preserved. B, Moreover, active bleeding (red arrow) was observed on the axial image scanned at the lower level, and there was no pneumoperitoneum or abnormal thickening of the duodenal wall (yellow arrow head). C, Follow-up MDCT scan 10 days after transcatheter arterial embolization (TAE) revealed a low-density lesion indicating an old hematoma (white arrow) at the posterior aspect of the pancreas head, and this lesion was smaller than before TAE. D, At 7 months follow-up computed tomography (CT), the hematoma had disappeared completely.

**Figure 2:**
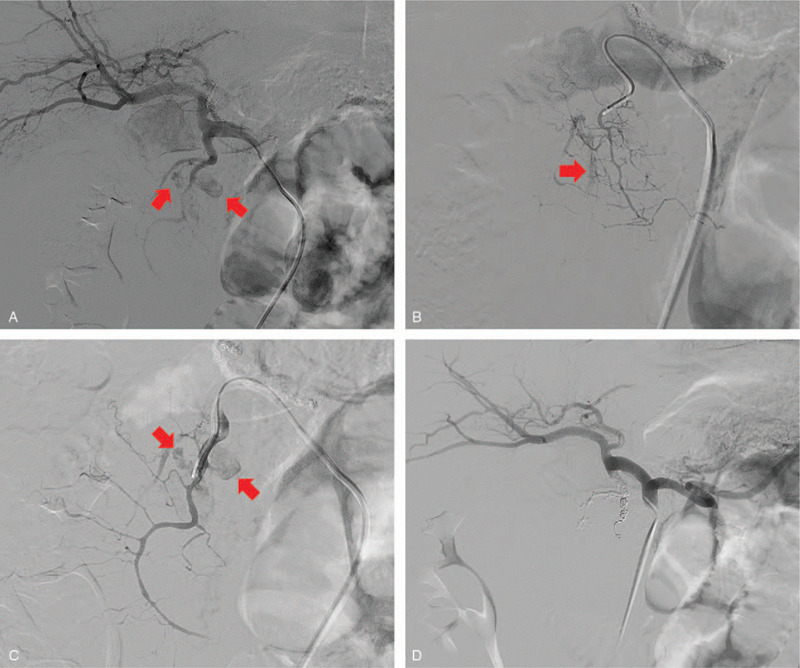
Case 1: a 64-year-old man was admitted to our trauma center after falling down from a height of 4 m. A, Common hepatic angiography showed multiple active bleedings (red arrows) from the superior pancreaticoduodenal artery (SPDA). B, Moreover, selective angiography using the microcatheter of the anterior superior pancreaticoduodenal artery (ASPDA) and (C) the posterior superior pancreaticoduodenal artery demonstrated (PSPDA) active bleedings (red arrows), the feeding arteries, and multiple fine vessels communicating with the active bleeding. D, The postembolization angiography demonstrated successful hemostasis with occlusion of the active bleeding.

A 64-year-old man was admitted to our trauma center after falling down from a height of 4 m. He had undergone subtotal gastrectomy (Billroth type I) for gastric cancer 4 years ago. At presentation, his vital signs were as follows: pulse, 62 beats/min and blood pressure (BP), 136/66 mm Hg. His only complaint was severe abdominal pain. Multidetector computed tomography (MDCT) scan revealed a large amount of retroperitoneal hematoma at the posterior aspect of pancreas head with internal extravasation of contrast media that suggested active bleeding (Fig. 1A and B). Although there was a subtle low-density lesion suggesting mild contusion at the head of the pancreas, the shape of pancreas and biliary duct were well-preserved (Fig. 1A), and no pneumoperitoneum or abnormal thickening of the duodenal wall was observed (Fig. 1B). To control the bleeding in the abdomen, TAE was attempted first, with the intention of performing surgical laparotomy in the case of worsening of the injuries in the pancreas or duodenum. A common hepatic artery angiography via the femoral approach revealed multiple active extravasations from the superior pancreaticoduodenal artery (Fig. 2A). Moreover, selective angiography of the anterior superior pancreaticoduodenal artery (ASPDA) (Fig. 2B) and posterior superior pancreaticoduodenal artery (PSPDA) (Fig. 2C) using the microcatheter demonstrated active bleedings. The tip of the microcatheter was advanced to the distal portion of the injured PSPDA and embolization was performed using a 4-fibered platinum microcoils. In contrast, given the ASPDA angiography finding (Fig. 2B), which showed the feeding arteries and multiple fine vessels communicating with the active bleeding, we decided to use a 1:2 mixture of *n*-butyl cyanoacrylate (NBCA) and iodized oil as an embolic agent. A postembolization angiography demonstrated successful hemostasis with the occlusion of the active bleedings (Fig. 2D), and collateral pancreaticoduodenal perfusion via inferior pancreaticoduodenal artery (IPDA) was found to be preserved on the superior mesenteric artery (SMA) angiography. The laparotomy was not performed because the patient remained hemodynamically stable without abdominal complications after TAE. The postoperative course was unremarkable, and the patient was discharged 1 month after the procedure. Follow-up computed tomography (CT) 10 days after TAE revealed a low-density lesion indicating an old hematoma at the posterior aspect of pancreas head (Fig. 1C), and the lesion was smaller than before TAE. In the 7 months follow-up CT, the hematoma had completely disappeared (Fig. 1D), and the patient had no abdominal symptoms.

### 2.2 Case 2

**Figure 3:**
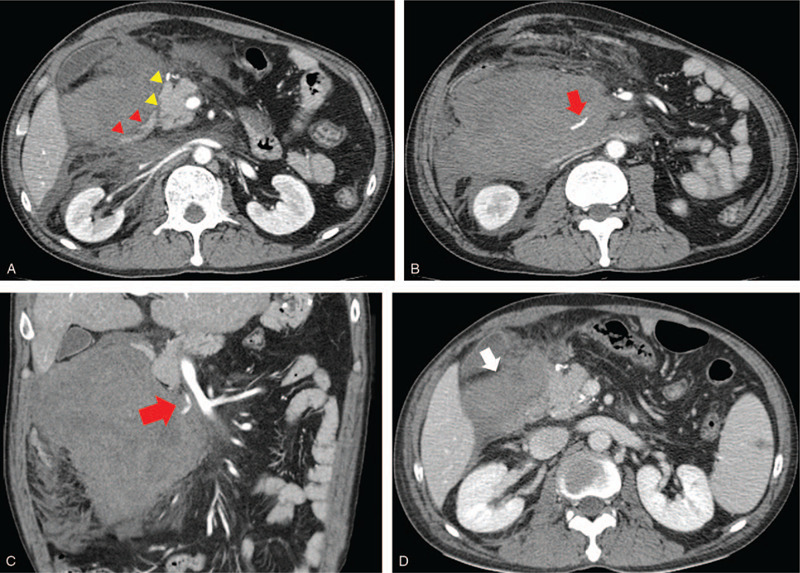
Case 2: a 45-year-old man was admitted to the trauma center after being hit by a car. A, An axial multidetector computed tomography (MDCT) scan revealed a large amount of retroperitoneal hematoma at the right lateral aspect of the pancreas head and second portion of the duodenum. Although they were compressed by a huge hematoma, the margin of the pancreas (yellow arrow head) and the duodenum (red arrow head) are well-preserved. B, An axial MDCT image scanned at the lower level showed active bleeding (red arrow) within the retroperitoneal hematoma. C, The coronal MDCT scan showed that the active bleeding (red arrow) was located between the pancreas head and the superior mesenteric vessels. D, Follow-up MDCT scan 7 days after transcatheter arterial embolization (TAE) revealed an old hematoma at the right lateral aspect of the pancreas head and it was smaller than before TAE.

**Figure 4:**
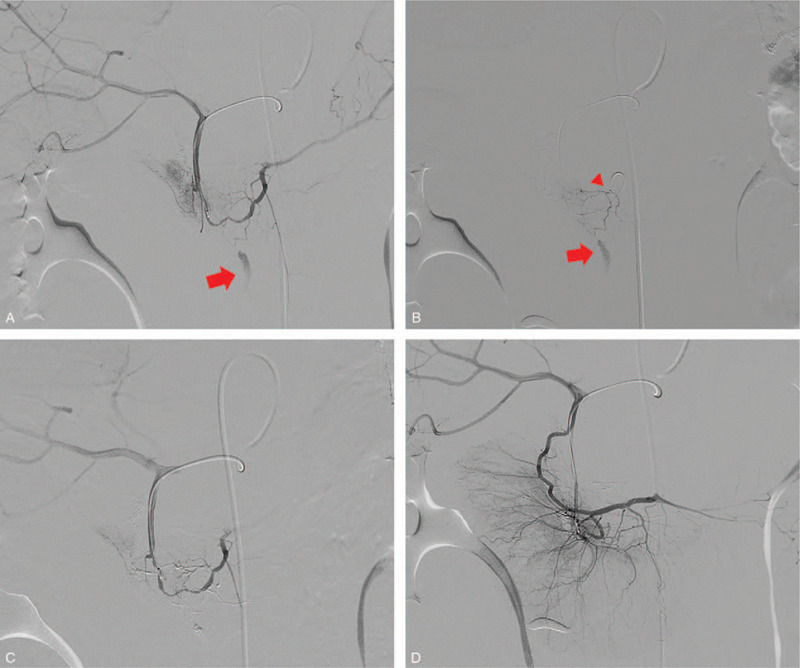
Case 2: a 45-year-old man was admitted to the trauma center after being hit by a car. A, Selective angiography of the inferior pancreaticoduodenal artery (PIPDA) via the gastroduodenal artery showed active bleeding (red arrow) from a branch of the PIPDA. B, Advancing the microcatheter to superselectively catheterize the bleeding site using the smallest caliber of microcatheter failed as the tip of the microcatheter (red arrow head) was actually apart from the involved artery (red arrow) due to the tortuosity and fine size of the artery. C, After transcatheter arterial embolization (TAE), a control angiography demonstrated complete occlusion of the bleeding, and the pancreaticoduodenal perfusion was preserved by collateral flow from the anterior inferior pancreaticoduodenal artery (AIPDA).

A 45-year-old man was admitted to the trauma center after being hit by a car. He had undergone subtotal gastrectomy (Billroth type II) for gastric cancer 3 years ago. His chief complaint was severe abdominal pain. On admission, his BP, pulse, and hemoglobin were 97/63 mm Hg, 160 beats/min, and 8.5 g/dL, respectively, and he received emergency packed red blood cell transfusions. The vital sign became stable after fluid resuscitation and transfusion. Subsequently, we performed MDCT, which revealed a large amount of retroperitoneal hematoma (Fig. 3A) at the right lateral aspect of the pancreas head with internal extravasation of the contrast media suggesting active bleeding (Fig. 3B). Although the head of the pancreas and the second portion of duodenum were compressed by the huge hematoma, the margin of pancreas and duodenum were well-preserved. Moreover, there were no injuries to the adjacent organs including kidney and liver and no evidence of pneumoperitoneum or abnormal thickening of intestine suggesting bowel injury. TAE was attempted immediately to control the active bleeding. Gastroduodenal artery (GDA) and SMA angiographies via the right femoral artery approach were performed, but the culprit artery was not clearly identified. Considering the CT finding in which the active bleeding was located between the pancreas head and superior mesenteric vessels (Fig. 3C), it was predicted that a branch such as the IPDA was the most likely bleeding source. Therefore, the microcatheter was advanced to the posterior IPDA (PIPDA) via the GDA, and selective angiography revealed active extravasation from the branch of the PIPDA (Fig. 4A). We were unable to advance the microcatheter to selectively catheterize the bleeding site using the smallest caliber of microcatheter (1.7 Fr) due to the tortuosity and fine size of the involved artery (Fig. 4B). Because NBCA is a highly penetrable liquid, a 1:2 mixture of NBCA and iodized oil was used as an embolic agent. After TAE, the control angiography demonstrated complete occlusion of the bleeding (Fig. 4C), and pancreaticoduodenal perfusion was preserved by collateral flow from the anterior IPDA (Fig. 4D). Follow-up CT 7 days after TAE revealed an old hematoma at the right lateral aspect of pancreas head (Fig. 3D), and this was smaller than before TAE. There was no evidence of complication, and the patient's vital signs had improved after TAE. The patient recovered and was discharged 8 weeks later.

### 2.3 Ethical considerations

The study design was approved by the Chungbuk National University Hospital Institutional Review Board, and the written informed consent was obtained from the 2 patients for publication of this case report and accompanying image.

## 3 Discussion

Pancreaticoduodenal trauma is rare but has a significant morbidity (36%–60%) and mortality (18%–23%).^[4]^ The outcome depends on early diagnosis, which is essentially based on a high index of suspicion. Management of pancreaticoduodenal trauma and isolated PDAH is problematic due to the complex anatomical relations of the duodenum, pancreas, biliary tract, and major vessels, and difficulties in the diagnosis and determination of the treatment strategies.^[3]^ Therefore, pancreaticoduodenal traumas are traditionally treated through surgeries.^[1]^ However, when the PDAH is not associated with duodenal perforation or advanced pancreatic injury with bile leakage, TAE can be used to treat hemorrhage and hemostasis as it is less invasive than laparotomy. Therefore, it is important to accurately determine whether surgery is required based on initial MDCT scan. MDCT is usually used as the initial diagnostic modality for the detection of the bleeding focus after blunt abdominal trauma.^[2]^ The sensitivity and specificity of MDCT scan in detecting pancreaticoduodenal trauma was reported to be as high as 91%.^[6]^ In our cases, the MDCT scans showed no specific or suggestive finding of bowel injuries including bowel wall discontinuity, extraluminal air, or high-grade pancreatic injury with ductal injury before and after TAE. Therefore, we attempted TAE as the first treatment to control the bleeding. In addition, a recent study reported that MDCT found active bleeding more frequently than angiography.^[5]^ Therefore, MDCT and angiography have complementary roles in the management of patients with pancreaticoduodenal trauma. Moreover, it may help the interventional radiologist to plan for the procedure. Thus, it is likely to decrease the number of angiograms required to localize the bleeding site. In our cases, MDCT facilitated the approximate localization of the bleeding site from the adjacent organ. In case 2, the initial MDCT revealed the active bleeding and the suspected branch of the hemorrhage site before angiography, which helped us to find the cause of PDAH which was a branch of the IPDA. Thus, MDCT would be the most useful diagnostic modality and should be performed immediately in hemodynamically stable patients with pancreaticoduodenal trauma.

Endovascular options may play an important role in the diagnosis and treatment of pancreaticoduodenal trauma. Angiography can detect and localize the involved artery, and TAE can effectively stop acute bleeding with a lower morbidity and mortality than surgery.^[7]^ Skilled interventional devices including microcatheter and embolic agents have increased the utility of TAE in various conditions. The choice of embolic agents is based on the vascular anatomy, angiographic findings, catheter position, and preferred operator. Embolization of the feeding artery was achieved using different combinations of embolic agents.^[7]^ In our cases, NBCA was used as an embolic agent in both cases. The use of NBCA has recently gained acceptance. NBCA is widely used for controlling active bleeding after trauma. It rapidly polymerizes with the blood, and therefore, it is advantageous for controlling massive hemorrhage that requires urgent hemostasis, especially in patients with coagulopathy.^[7]^ In both our cases, successful embolization was achieved, and the patients were discharged without recurrent bleeding after embolization with NBCA. This result may be explained by 2 factors. First, NBCA achieved complete embolization of the target artery and filled the adjacent potential collateral vessels. Therefore, complete embolization is expected in PDAH with multiple collateral vessels. Second, other potential advantages of NBCA include its capacity to obliterate small distal vessels because of its highly penetrable and liquid nature. In most patients, PDAH is likely to originate from a small-sized branch artery with a severe tortuous anatomy, similar to case 2. In such cases, NBCA can be considered as a useful material for the embolization of PDAH. In addition, TAE for the PDAH can increase the risk of duodenal ischemia by reducing the blood flow to the bowel segment, and moreover, nontargeted embolization can occur.^[8]^ However, in the past decade, the advanced superselective catheterization technique has helped to decrease risk. In our cases, preservation of the affected duodenum and pancreas perfusion by collateral flow were identified on final angiograms after embolization with NBCA, and therefore, there were no duodenal infarctions or pancreatitis. These results showed that embolization with the use of NBCA is technically feasible, safe, and effective for the treatment of traumatic PDAH.

**Figure 5:**
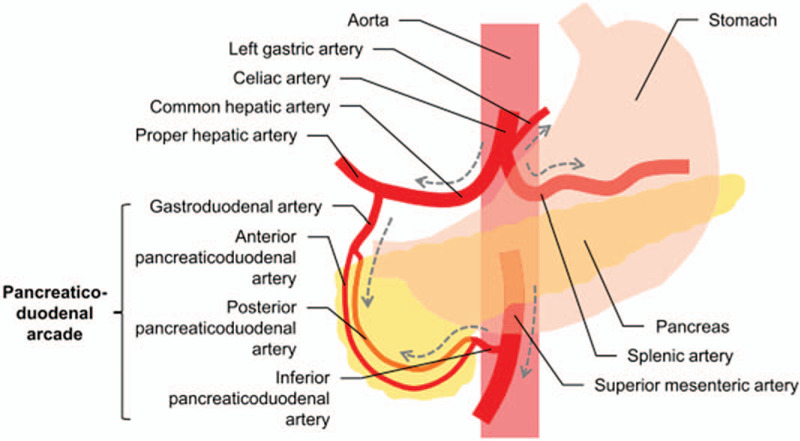
Normal anatomy of the visceral arteries branching from the celiac and superior mesenteric arteries. The arrows indicated the flow directions under normal conditions.

In pancreaticoduodenal trauma, hemorrhage may result from injury to PDA, which has an abundant vascular supply and multiple potential collateral channels (Fig. 5). These arteries form connections or anastomoses with one another, allowing blood to perfuse the head of pancreas and duodenum through multiple channels.^[3]^ Therefore, an understanding of the possible multifocality in bleeding due to pancreaticoduodenal trauma is important because it could be the cause of treatment failure of TAE. In case 1, multifocal active bleedings were revealed in both ASPDA and PSPDA angiography, and feeding arteries and multiple fine vessels communicating with the active bleedings were identified. Therefore, complete embolization with NBCA should be performed simultaneously on both feeding arteries and multiple fine vessels. In case 2, multiple selective angiographies were performed on the SMA, celiac trunk, and GDA which identified the absence of involved artery. This false negative finding may be explained by the back flow caused by abundant collateral supply. Fortunately, stagnation of contrast media suggesting active bleeding was successfully identified after the microcatheter was fully advanced to the proximal portion of the PIPDA and by increasing the volume of contrast medium on the angiogram.

For a successful TAE, definite identification of the causative artery, selective catheterization of the feeding arteries and complete embolization are a prerequisite. Therefore, we recommend that the following points be considered when TAE is performed in patients with hemorrhage after pancreaticoduodenal trauma. First, when performing angiography, maximum amount of contrast media should be used and selective angiography using microcatheter should be performed whenever possible. Second, considering the abundant vascular supply and multiple potential collateral channels of PDA, it is highly recommended to perform complete angiography of the GDA and SMA at the initial session and to use NBCA as the embolic agent for complete embolization.

In conclusion, patients with PDAH following blunt abdominal trauma require prompt clinical and imaging evaluation. Therapeutic angiography is emerging as an effective and safe treatment strategy and minimally invasive alternative to surgery for the management of isolated PDAH without any sign of duodenal perforation or advanced pancreatic injury. It is expected that a carful and repetitive approach, based on complete angiography and embolization with NBCA, could increase the success rate of TAE.

## Author contributions

**Conceptualization:** Yook Kim, Jisun Lee, Bum S. Cho.

**Data curation:** Yeongtae Park, Yook Kim.

**Formal analysis:** Yeongtae Park, Yook Kim.

**Resources:** Yook Kim, Jin Y. Lee.

**Supervision:** Yook Kim, Jisun Lee, Bum S. Cho.

**Writing – original draft:** Yeongtae Park, Yook Kim.

**Writing – review & editing:** Yeongtae Park, Yook Kim.
