# Pancreaticoduodenal arterial hemorrhage following blunt abdominal trauma treated with transcatheter arterial embolization: Two case reports: Erratum

**DOI:** 10.1097/MD.0000000000032716

**Published:** 2023-01-13

In the article, “Pancreaticoduodenal arterial hemorrhage following blunt abdominal trauma treated with transcatheter arterial embolization: Two case reports,”^[1]^ which publish in Volume 99, Issue 40 of *Medicine*, publication permission for Figure 5 was not included and should have appeared as:

Yuhn C, Hoshina K, Miyahara K, Oshima M. Computational simulation of flow-induced arterial remodeling of the pancreaticoduodenal arcade associated with celiac artery stenosis. Journal of Biomechanics. 2019;92:146-154.

https://doi.org/10.1016/j.jbiomech.2019.05.043

## References

[1] Park Y Kim Y Lee J. Pancreaticoduodenal arterial hemorrhage following blunt abdominal trauma treated with transcatheter arterial embolization: two case reports. Medicine (Baltimore). 99:e22531. 10.1097/MD.0000000000022531 PMC7535771 33019457

